# Supplementary material for: The force required to remove tubulin from the microtubule lattice by pulling on its α-tubulin C-terminal tail
Source: Nat Commun. 2022 Jun 25;13:3651. doi: 10.1038/s41467-022-31069-x (PMC9233703; doi:10.1038/s41467-022-31069-x)
Supplement: Supplementary file 1 — Supplementary information [file 41467_2022_31069_MOESM1_ESM.pdf]

Supplementary Information

The force required to remove tubulin from the microtubule lattice by pulling on its α-tubulin C-terminal tail

Yin-Wei Kuo, Mohammed Mahamdeh, Yazgan Tuna, Jonathon Howard*

*Corresponding author: Jonathon Howard

Email: joe.howard@yale.edu

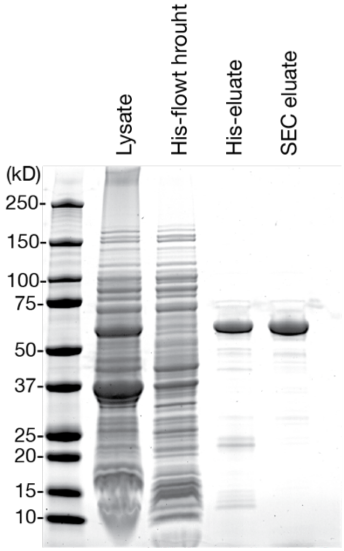

Supplementary Figure 1. SDS-PAGE of purification steps of recombinant tubulin tyrosine ligase (TTL). Human TTL with N-terminal His_6-SUMO tag was expressed in E. coli and purified by HisTrap affinity column and size exclusion chromatography (SEC). Predicted molecular weight of His_6-SUMO-TTL: 57 kD. Purification of TTL was repeated once.

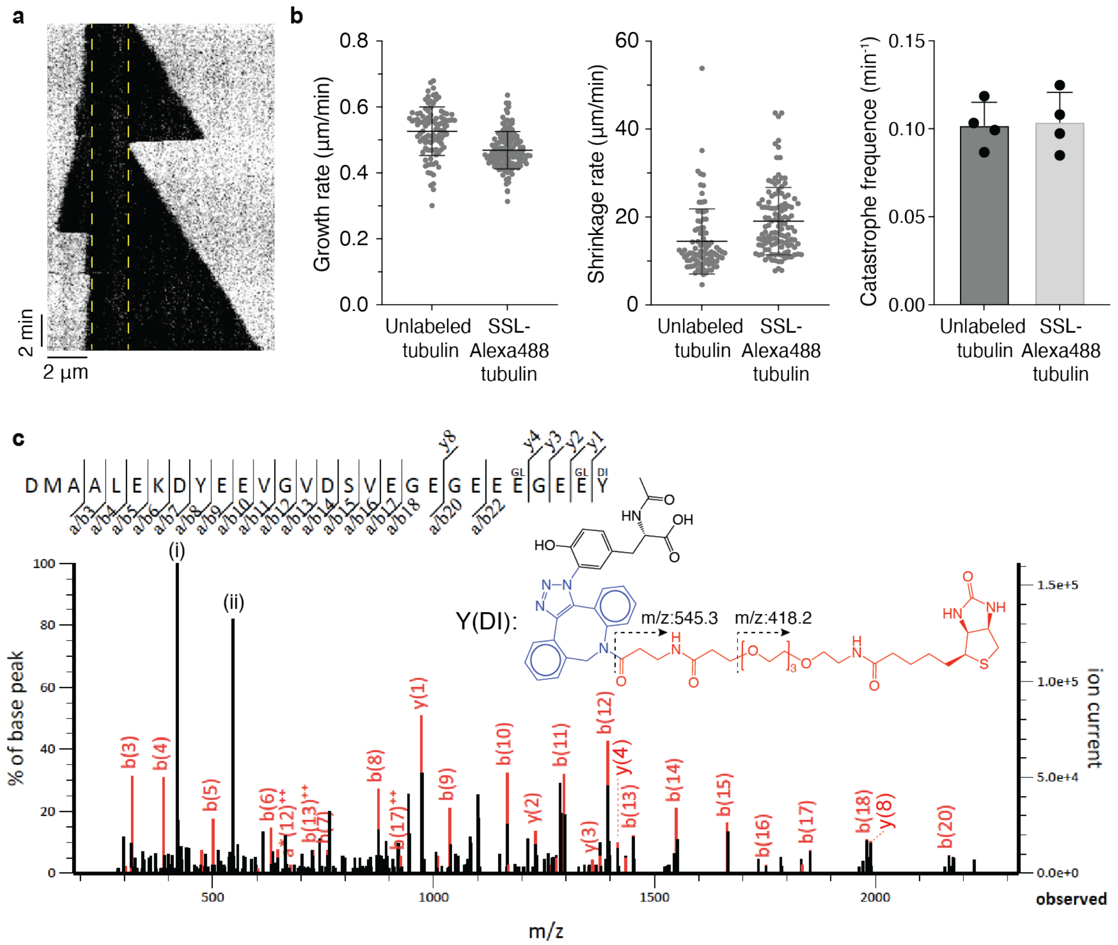

Supplementary Figure 2. TTL-dependent site-specific labeling had little effects on microtubule dynamics. a Example kymograph of a dynamic microtubule extension growing off a GMP-CPP stabilized microtubule seed polymerized from 8 µM Alexa Fluor 488-conjugated tubulin using the TTL-dependent site-specific labeling (SSL) method imaged by IRM. The seed position is marked by yellow dashed lines. **b** Comparison of the dynamic properties of 8 µM unlabeled and SSL-Alexa Fluor 488-tubulin (labeling density 43%) imaged by IRM from four independent measurements. Dynamic parameters of unlabeled vs. SSL-tubulin (mean ± SD): growth rates (0.53 ± 0.07 vs. 0.47 ± 0.06 µm/min; *n* = 99 vs. 131 events); shrinkage rate (14 ± 7 vs. 19 ± 8 µm/min; *n* = 89 vs. 119 events); catastrophe frequency (0.102 ± 0.013 vs. 0.104 ± 0.008 min^{-1}; *n* = 4 experiments). **c** Example MS/MS spectrum of α-tubulin C-terminal peptide conjugated to biotin via the site-specific labeling method. Y(DI) shows the [3+2] cycloaddition product on the C-terminal tyrosine and was used as custom modification in the MASCOT search. Mono- and polyglutamate chains (GL) up to 4 glutamate residues were included as possible PTMs in the MASCOT search as well. The detected ion fragments corresponding to the cleavage of backbone bonds are highlighted in red. Note that two reporter ions (peak i and ii) corresponded to the fragmentation at the linker (dashed arrows).

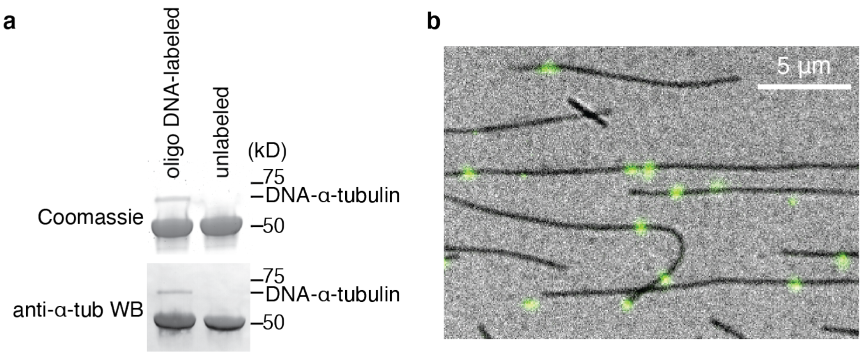

Supplementary Figure 3. Incorporation of oligo-DNA handle and long DNA linkers. a Successful conjugation of oligo-DNA to α-tubulin via the site-specific labeling method was confirmed by the molecular weight shift of the oligo-DNA-labeled tubulin. The labeling was performed with triplicates. **b** Example of taxol-stabilized microtubule with long DNA-linkers (green puncta). The DNA linkers were stained with SYTOX Green and imaged by TIRF microscopy. The microtubules were imaged by IRM. The experiments were repeated with duplicates.

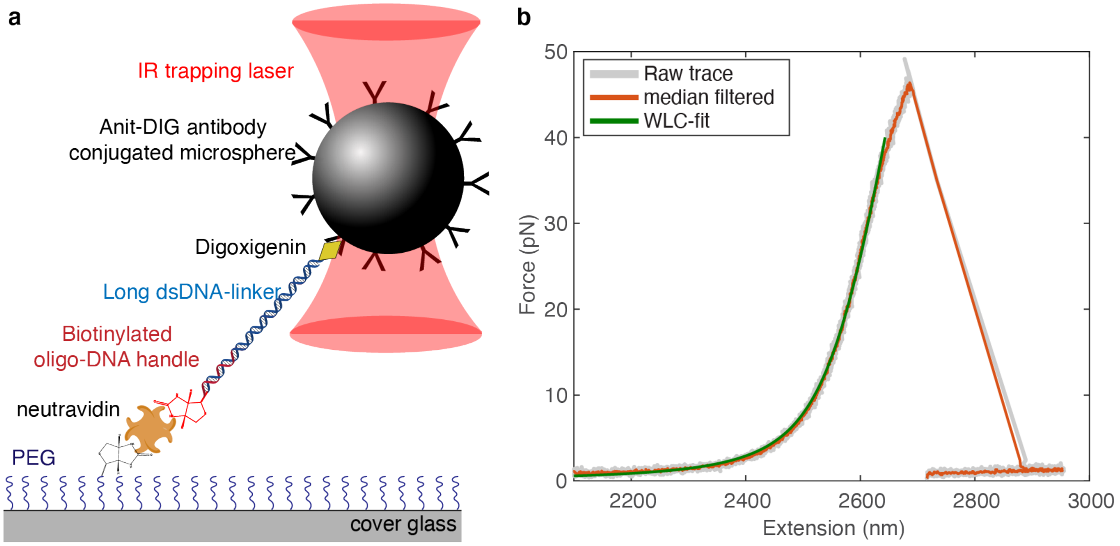

Supplementary Figure 4. Surface-tethered DNA linker pulling controls. a Experimental scheme of the surface-tethered DNA control setup. The DNA linkers were first hybridized to the biotinylated oligo-DNA handle (identical sequence as the handle used in the tubulin pulling experiments). The coverslips were first covalently biotinylated followed by binding of neutravidin. The DNA-linkers hybridized to biotinylated DNA oligo were then anchored onto the surface via biotin-neutravidin binding. Note that the same DNA linker and microspheres were used in both the control experiment and the taxol-microtubule pulling experiments. **b** Example force extension curve of stretching surface-tethered DNA linker.

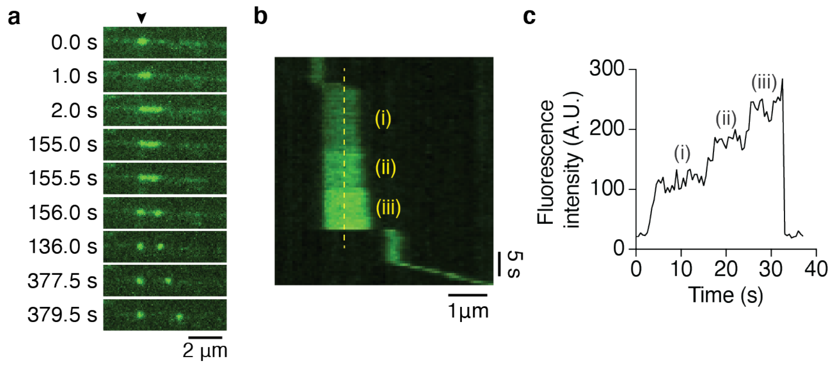

Supplementary Figure 5. Examples of photocleavage and step-wise force increase in the kinesin pulling assay. a Example time-lapse images of a DNA photocleavage event after stretched due to photo-induced double-stranded break. The anchor point is indicated by the arrowhead. **b, c** Example kymograph and fluorescence intensity line-scan (yellow dashed line in **b**) showing step-wise increase of DNA-fluorescence intensity during the stretching phase. Three distinct steps can be identified from the intensity trace in **c**. The increase of intensity corresponded to the increase of pulling force on the DNA[1]. The step-wise increase of force was likely due to the increase number of kinesin molecules engaging in the force generation process. A.U.: analog digital unit.

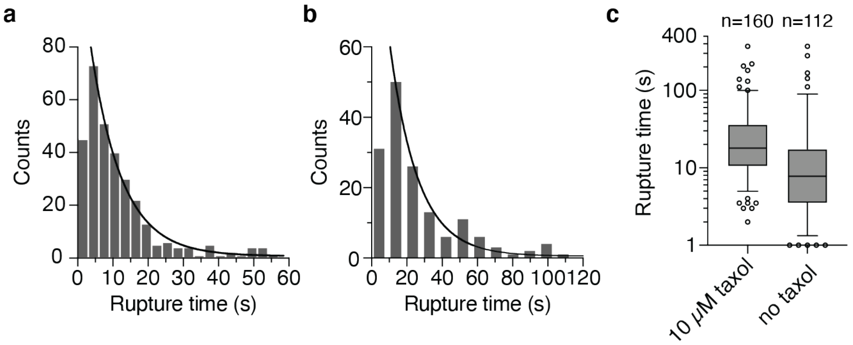

Supplementary Figure 6. Rupture time from kinesin assays. a Rupture time histogram of kinesin-pulling assays without taxol (pooled from 4.5 to 9 nM; total 333 events; ~95% of data is within 0 to 60 s and was plotted here). The histogram was fitted with a single exponential decay (black line) with the first bin left out of the fit due to the difficulty of measuring short rupture time (< 3 s). Decay time of exponential fit was 8.8 s; $R^2 = 0.988$. **b** Rupture time histogram of kinesin-pulling assays with taxol microtubules (6 nM of kinesin-biotin; total 160 events; only the first 120s were shown). Decay time of exponential fit (with the first bin left out) was 15.9 s; $R^2 = 0.97$. **c** Rupture time of taxol microtubules is longer than the non-taxol-stabilized microtubules (GDP-MT) in the motor pulling assay (Mann-Whitney U test, $p<0.0001$). Taxol: median=18.0s, 95% CI [14.5s, 22.0s]; no taxol: median=7.8 s, 95% CI [6.0s,10.0s]; n: number of events. Box plot: 25th, 75th percentile; center line: median; whisker: 5th and 95th percentile. Both experiments were performed with 6 nM kinesin-biotin. **d** The rupture time (from the GDP-microtubule pulling assay) where the

DNA was stretched only once or multiple times showed no significant difference (Mann-Whitney U test, p=0.24). This suggests there is no evident memory effect for the tubulin extraction, potentially due to the rapid refolding (see Appendix below). n: number of events. Box plot: 25th, 75th percentile; center line: median; whisker: 5th and 95th percentile. The data were pooled from saturating concentration of biotinylated kinesin (4.5 to 9 nM).

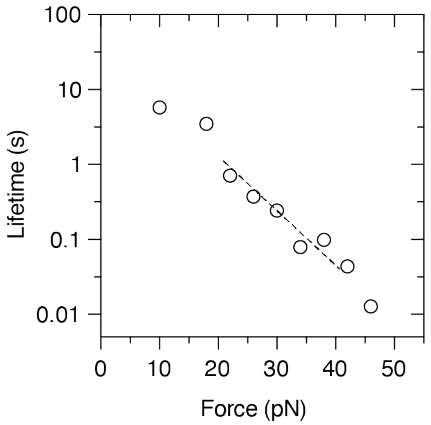

Supplementary Figure 7. Estimated force-dependent rupture time. Force-dependent rupture time τ(*F*) was estimated from the rupture force histogram obtained in the taxol-stabilized microtubule pulling assay using optical tweezer in Fig. 3C by applying the method described in [2]. Fit with Bell model τ_0 = 37.1 ± 1.5 s, $x^\ddagger$ = 0.69 ± 0.05 nm; R^2 = 0.96. Note that this is an underestimation of the true force-dependent rupture time of tubulin subunit since the DNA-bead rupture limit the maximum force that can be measured.

Supplementary Table 1. Primer pairs for DNA linker preparation

Primer 1	Primer 2	DNA linker size (kb)
5'-TCTAAGTGACGGCTGCATACTAACC-3' (5' end and underlined bases were labeled with digoxigenin)	5'-CGCCTGCGTAGGATATCGCAGATACCGCATCAGTCCAXCAACGGTCGATTGCCTGACGGA-3' (X: abasic)	8.2
5'-biotin-GCCAATGCGCTTACTGATGCGG-3'	5'-CGCCTGCGTAGGATATCGCAGATACCGCATCAGTCCAXGGTTTCACTGCTGGCGTATGACC(X: abasic)	3.8

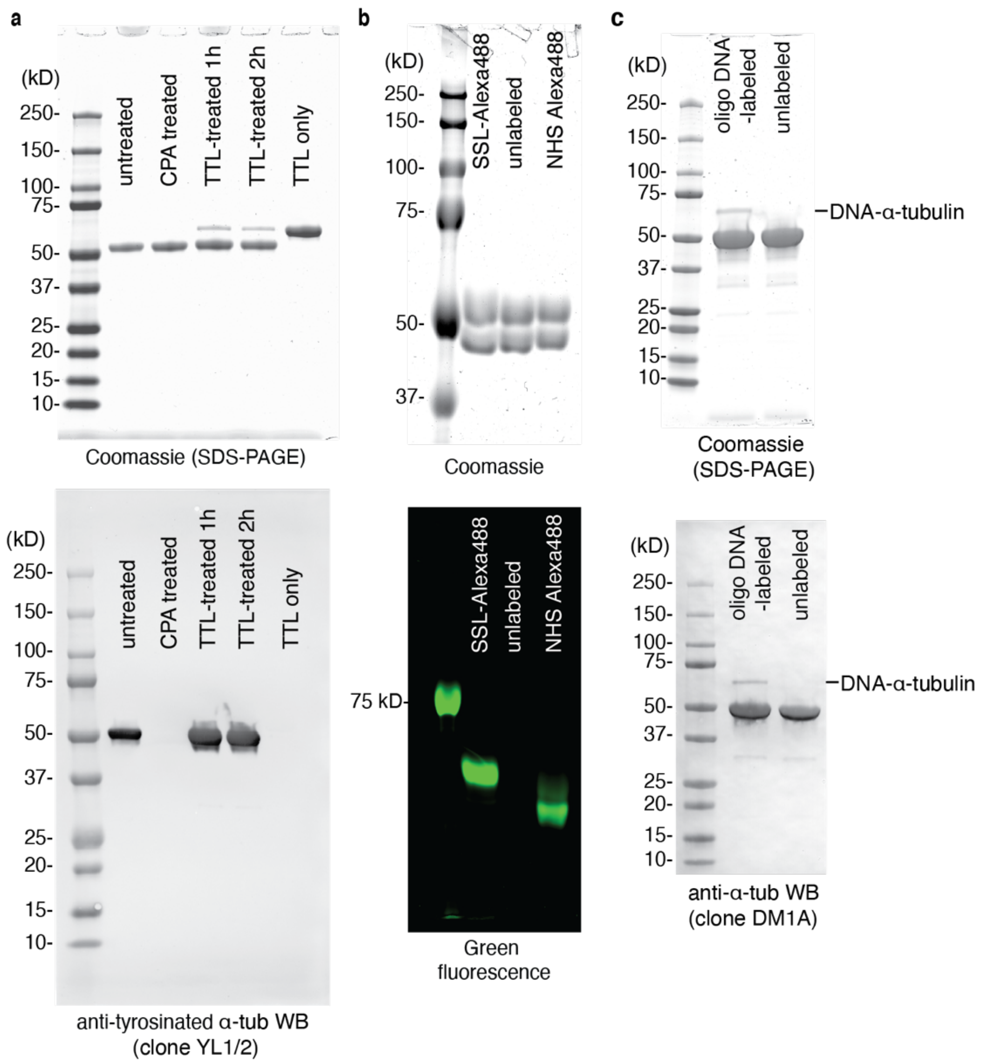

Supplementary Figure 8. Uncropped gels and western blots. a Uncropped SDS-PAGE gel and western blot for the tyrosination assay in Fig.1A. **b** Uncropped high resolution SDS-PAGE of tubulin corresponding to Fig.1C. **c** Uncropped SDS-PAGE of oligo DNA-labeled tubulin in Fig.S3A.

Appendix: two-step tubulin extraction model

We consider a simple two-step pathway for the dissociation of the lattice-bound tubulin (A) to the unbound state (C). Suppose that the removal of tubulin from the lattice takes place through a partially unfolded intermediate (B) that can refold at low force. The energy landscape is illustrated below (Figure S9).

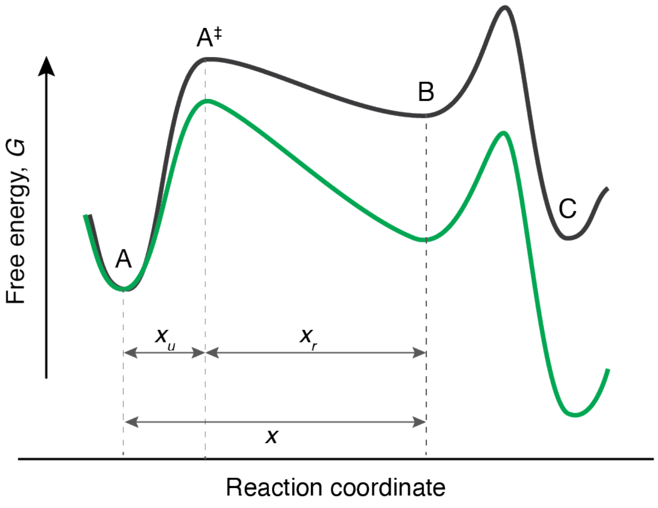

Supplementary Figure 9. Schematic diagram of the free energy landscape of a two-step tubulin removal pathway. Black line indicates the energy landscape under no force. In the presence of external mechanical force, the energy landscape is tilted (green). A: lattice-bound state; $A^{\ddagger}$: transition state; B: partially unfolded intermediate; C: unbound tubulin (dissociates from the lattice).

We can write the reaction as:

$$\mathrm{A} \underset{k_{\mathrm{r}}}{\overset{k_{\mathrm{u}}}{\rightleftharpoons}} \mathrm{B} \overset{k_{BC}}{\rightarrow} \mathrm{C}$$

We consider the extraction step ($\mathrm{B} \to \mathrm{C}$) to be essentially irreversible; the tubulin does not rebind once it is removed from the lattice. In the absence of external force, $G_B \gg G_A$, so the intermediate B rapidly refolds and the spontaneous unfolding rate is very low. Force tilts the energy landscape (green curve in Figure S9), decreasing $G(\mathrm{B})$ more than $G(A^{\ddagger})$; therefore the unfolding ($\mathrm{A} \to \mathrm{B}$) rate increases while the refolding ($\mathrm{B} \to \mathrm{A}$) is inhibited. However, proteins are quite “brittle” (Howard 2001[3], Example 5.3) so the strain of the transition state $A^{\ddagger}$ (x_{u}) is smaller than the total extension of the unfolded intermediate ($x_{\mathrm{u}} < x_{\mathrm{u}} + x_{\mathrm{r}}$): it only takes a small extension to unfold a protein, which then elongates considerably. Therefore, the refolding is more force dependent.

This picture can be formalized by modeling the force-dependent rate constants of unfolding $k_{\mathrm{u}}(F)$ and refolding $k_{\mathrm{r}}(F)$ using the Bell formula [4]:

$$k_{\mathrm{u}}(F) = \frac{1}{\tau_{\mathrm{u}}^{0}} \exp\left(\frac{Fx_{\mathrm{u}}}{kT}\right)$$
$$k_{\mathrm{r}}(F) = \frac{1}{\tau_{\mathrm{r}}^{0}} \exp\left(-\frac{Fx_{\mathrm{r}}}{kT}\right)$$

where τ_{u}^{0} and τ_{r}^{0} are respectively the lifetimes of the unfolding and refolding reactions under no force. At steady state, $d\mathrm{B}/\mathrm{dt} = 0$ and the tubulin extraction rate r_{ex} is expressed as:

$$r_{\mathrm{ex}} = -\frac{1}{A}\frac{dC}{dt} = -\frac{1}{A}k_{\mathrm{BC}}B = \frac{k_{\mathrm{u}}}{1 + k_{\mathrm{r}}/k_{\mathrm{BC}}}$$

Thus, the overall lifetime of a lattice-bound tubulin $\tau(F)$ is:

$$\tau(F) = \frac{1}{k_{\mathrm{u}}}(1 + k_{\mathrm{r}}/k_{\mathrm{BC}}) = \tau_{\mathrm{u}}^{0} \exp\left(-\frac{Fx_{\mathrm{u}}}{kT}\right)\left[1 + (\tau_{\mathrm{BC}}/\tau_{\mathrm{r}}^{0}) \exp\left(-\frac{Fx_{\mathrm{r}}}{kT}\right)\right]$$

Where $\tau_{\mathrm{BC}} = 1/k_{\mathrm{BC}}$. For simplicity we assume that the second reaction step ($\mathrm{B} \rightarrow \mathrm{C}$) is force independent. However, it is expected that external force will accelerate this reaction step as well, but this does not change the main conclusion.

When the external force is large, $\tau(F) \rightarrow \tau_{\mathrm{u}}^{0} \exp\left(-\frac{Fx_{\mathrm{u}}}{kT}\right)$ and the rate-limiting step is the unfolding step. From the Dudko analysis of the unfolding force histogram (Fig. 4d), we can estimate that $\tau_{\mathrm{u}}^{0} \sim$ 30 sec and x_{u} ~1 nm. The lifetime without external force $\tau(0)$ is much longer: $\tau(0) = \tau_{\mathrm{u}}^{0}\left(1 + \frac{\tau_{\mathrm{BC}}}{\tau_{\mathrm{r}}^{0}}\right)$, which can be arbitrarily large if $\tau_{\mathrm{BC}} \gg \tau_{\mathrm{r}}^{0}$. For the taxol-stabilized microtubules, we expect that $\tau(0)$ to be very large, perhaps on the order of 10^6 s or longer, because they are typically stable for hours to days (a spontaneous lifetime of 10^6 s corresponds to ~1 spontaneous event per micrometer per 10 minutes). If we estimate $\tau(0) \sim$ 10^6 s and $x_{\mathrm{r}} \sim 5\ \mathrm{nm}$ (~5 times longer than x_{u}), we can estimate the force that corresponds to a lifetime of ~18 s in the taxol motor-pulling assay (Figure 5e, S6c) to be ~8 pN, consistent to the stall force of one to two kinesin motors. Other combinations of the free parameters $\tau_{\mathrm{BC}}/\tau_{\mathrm{r}}^{0}$ and x_{r} can be chosen to simultaneously make the zero-force lifetime almost arbitrarily long and also $\tau(8\ \mathrm{pN})$ ~18 s.

In conclusion, with an intermediate unfolded state, the Bell curve for tubulin extraction from the lattice (Figure S7) can be extrapolated with an arbitrary exponential at low forces to account for the slow spontaneous dissociation of tubulin from the lattice shaft.

Supplementary Information References:

1. King, G. A., Biebricher, A. S., Heller, I., Peterman, E. J. G. & Wuite, G. J. L. Quantifying Local Molecular Tension Using Intercalated DNA Fluorescence. *Nano Lett.* **18**, 2274–2281 (2018).
2. Dudko, O. K., Hummer, G. & Szabo, A. Theory, analysis, and interpretation of

single-molecule force spectroscopy experiments. *Proc. Natl. Acad. Sci.* **105**, 15755–15760 (2008).

3. Howard, J. *Mechanics of Motor Proteins and the Cytoskeleton*. (Sinauer Associates Incorporated, 2001).
4. Bell, G. I. Models for the Specific Adhesion of Cells to Cells. *Science.* **200**, 618–627 (1978).
