# Supplementary material for: The force required to remove tubulin from the microtubule lattice by pulling on its α-tubulin C-terminal tail
Source: Nat Commun. 2022 Jun 25;13:3651. doi: 10.1038/s41467-022-31069-x (PMC9233703; doi:10.1038/s41467-022-31069-x)
Supplement: Supplementary file 3 — Description of additional Supplementary File [file 41467_2022_31069_MOESM3_ESM.pdf]

Descriptions of Additional Supplementary Data files

Supplementary movie 1: Example DNA-stretching and removal of tubulin from GDPmicrotubule lattice by motors. Corresponding kymograph and time-lapse images were shown in Fig. 5b, c.

Supplementary movie 2: Example of stepwise fluorescence increase of DNA when stretched by motors. The corresponding kymograph was shown in Figure 5f.
