# Supplementary material for: The force required to remove tubulin from the microtubule lattice by pulling on its α-tubulin C-terminal tail
Source: Nat Commun. 2022 Jun 25;13:3651. doi: 10.1038/s41467-022-31069-x (PMC9233703; doi:10.1038/s41467-022-31069-x)
Supplement: Supplementary file 6 — Reporting Summary [file 41467_2022_31069_MOESM6_ESM.pdf]

nature portfolio

Corresponding author(s): Jonathon Howard
Last updated by author(s): 05/24/2022

Reporting Summary

Nature Portfolio wishes to improve the reproducibility of the work that we publish. This form provides structure for consistency and transparency in reporting. For further information on Nature Portfolio policies, see our Editorial Policies and the Editorial Policy Checklist.

Statistics

For all statistical analyses, confirm that the following items are present in the figure legend, table legend, main text, or Methods section.

n/a	Confirmed	
☐	☒	The exact sample size (*n*) for each experimental group/condition, given as a discrete number and unit of measurement
☐	☒	A statement on whether measurements were taken from distinct samples or whether the same sample was measured repeatedly
☐	☒	The statistical test(s) used AND whether they are one- or two-sided<br>*Only common tests should be described solely by name; describe more complex techniques in the Methods section.*
☒	☐	A description of all covariates tested
☒	☐	A description of any assumptions or corrections, such as tests of normality and adjustment for multiple comparisons
☐	☒	A full description of the statistical parameters including central tendency (e.g. means) or other basic estimates (e.g. regression coefficient) AND variation (e.g. standard deviation) or associated estimates of uncertainty (e.g. confidence intervals)
☐	☒	For null hypothesis testing, the test statistic (e.g. *F*, *t*, *r*) with confidence intervals, effect sizes, degrees of freedom and *P* value noted<br>*Give P values as exact values whenever suitable.*
☒	☐	For Bayesian analysis, information on the choice of priors and Markov chain Monte Carlo settings
☒	☐	For hierarchical and complex designs, identification of the appropriate level for tests and full reporting of outcomes
☒	☐	Estimates of effect sizes (e.g. Cohen's *d*, Pearson's *r*), indicating how they were calculated

Our web collection on statistics for biologists contains articles on many of the points above.

Software and code

Policy information about availability of computer code

Data collection	Optical tweezer data was collected by using Labview 2014 (National Instrument) as previously described (Schäffer et al. 2007 Langmuir); the microscopy data were collected by Nikon NIS element software version 5.11.03
Data analysis	Analysis of optical tweezer data was performed by custom written code using Matlab (2016b) and Labview (2014); Fiji version 2.0.0 was used for all image analysis; MASCOT version 2.4.0 was used for MS/MS spectra search.

For manuscripts utilizing custom algorithms or software that are central to the research but not yet described in published literature, software must be made available to editors and reviewers. We strongly encourage code deposition in a community repository (e.g. GitHub). See the Nature Portfolio guidelines for submitting code & software for further information.

Data

Policy information about availability of data

All manuscripts must include a data availability statement. This statement should provide the following information, where applicable:

- Accession codes, unique identifiers, or web links for publicly available datasets
- A description of any restrictions on data availability
- For clinical datasets or third party data, please ensure that the statement adheres to our policy

The source data presented in all figures are included in the source data file. Additional data supporting the findings of this study are available from the corresponding author upon request.

Field-specific reporting

Please select the one below that is the best fit for your research. If you are not sure, read the appropriate sections before making your selection.

- [x] Life sciences
- [ ] Behavioural & social sciences
- [ ] Ecological, evolutionary & environmental sciences

For a reference copy of the document with all sections, see nature.com/documents/nr-reporting-summary-flat.pdf

Life sciences study design

All studies must disclose on these points even when the disclosure is negative.

Sample size	Sample size calculation was not performed. The sample sizes of microscopy experiments (3 to 4 replicates) and optical tweezer traces (30-50 traces) are within standard in the field (Kuo et al. 2019 PNAS,Trushko et al. 2013 PNAS). Qualitative experiments that test the robustness of the site-specific labeling method were repeated at least with duplicates to confirm the reproducibility of the results.
Data exclusions	Optical tweezer traces and TIRF events were excluded based on the pre-determined criteria described in the materials and methods section. Traces deviated from the known force-extension curve of DNA were excluded; TIRF assay events whose rupture time cannot be determined correctly (i.e. the start of the events took place before image acquisition or stretched DNA overlapped with each other) were excluded from the analysis.
Replication	All experiments were performed over several months on multiple independent experimental days with success. All replication were included in the analysis and the relevant statistical parameters are presented.
Randomization	Randomization is not relevant for this study, as there was no need to distribute the samples into different experimental groups.
Blinding	Blinding was not possible in this study as a single individual performed all experiments and analyses. Pre-established analysis standard were used to reduce human bias.

Reporting for specific materials, systems and methods

We require information from authors about some types of materials, experimental systems and methods used in many studies. Here, indicate whether each material, system or method listed is relevant to your study. If you are not sure if a list item applies to your research, read the appropriate section before selecting a response.

Materials & experimental systems

n/a	Involved in the study
[ ]	[x] Antibodies
[x]	[ ] Eukaryotic cell lines
[x]	[ ] Palaeontology and archaeology
[x]	[ ] Animals and other organisms
[x]	[ ] Human research participants
[x]	[ ] Clinical data
[x]	[ ] Dual use research of concern

Methods

n/a	Involved in the study
[x]	[ ] ChIP-seq
[x]	[ ] Flow cytometry
[x]	[ ] MRI-based neuroimaging

Antibodies

Antibodies used	Anti-tyrosinated tubulin antibody: clone YL1/2, EMD-millipore, catalog number #MAB1864, 1:2500 dilution; anti-digoxigenin Fab fragment: Roche, catalog number #11214667001; anti-digoxigenin antibody: polyclonal, Roche, catalog number #11333089001; anti-alpha tubulin antibody: clone DMA1A, EMD Millipore, catalog number #MABT205, 1:5000 dilution; rabbit anti-rat secondary antibody (AP-conjugated): polyclonal, Invitrogen, catalog number # PA1-28787, 1:2000 dilution; goat anti-mouse secondary antibody (AP-conjugated): polyclonal, Invitrogen, catalog number # G-21060, 1:5000 dilution.
Validation	Specificity of all primary antibodies were provided by the manufacturers. Controls for tyrosinated tubulin antibody were included in the experiments (negative control: CPA-treated tubulin; positive control: untreated bovine brain tubulin)<br>Specificity statements from the manufacturers:<br>Anti-tyrosinated tubulin antibody, clone YL1/2: Reacts with alpha-tubulin with phenylalanine (yeast) or tyrosine (mammalian and chicken) at the C-terminus (PMID 6204858). Does not react toward posttranslationally modified alpha-tubulin lacking the C-terminus Phe or Tyr.<br>Anti-digoxigenin Fab and antibody: polyclonal antibody from sheep is specific to digoxigenin and digoxin and shows no cross-reactivity with other steroids, such as human estrogens and androgens.<br>Anti-alpha tubulin antibody, clone DMA1A:Recognizes α-Tubulin, clone DM1A, MW 50-60 kDa. The epitope recognized by this antibody has been mapped to amino acids 426-450<br>Rabbit anti-rat secondary antibody:affinity-purified antibodies with well-characterized specificity for rat immunoglobulins; detects Rat IgG from rat samples.

Goat anti-mouse secondary antibody: affinity-purified antibodies with well-characterized specificity for mouse immunoglobulins
